# ZnO Nanostars Decorated with Mass-Selected Au Clusters for Photoluminescence

**DOI:** 10.1021/acsanm.5c02913

**Published:** 2025-09-03

**Authors:** Gisella Di Mari, Giacometta Mineo, Henry Hoddinott, Vincenzina Strano, Claudio Lentini Campallegio, Bernat Mundet, Sara Martì Sanchez, Giorgia Franzò, Jordi Arbiol, Bernd Von Issendorff, Georg Held, Richard Palmer, Elena Bruno, Salvo Mirabella, Maria Chiara Spadaro

**Affiliations:** † Dipartimento di Fisica e Astronomia “Ettore Majorana”, Università di Catania, via S. Sofia 64, Catania 95123, Italy; ‡ IMM-CNR, Sede Catania Università, Via S. Sofia 64, 95123 Catania, Italy; § Nanomaterials Lab, Mechanical Engineering, 7759 Swansea University, Bay Campus, Fabian Way, Swansea SA1 8EN, U.K.; ∥ Diamond Light Source, Harwell Science and Innovation Campus, Didcot OX11 0DE, U.K.; ⊥ Catalan Institute of Nanoscience and Nanotechnology (ICN2), CSIC and BIST, Campus UAB, Barcelona 08193, Spain; # ICREA, Pg. Lluís Companys 23, Barcelona, Catalonia 08010, Spain; ∇ Department of Physics, Albert-Ludwigs-Universität, Freiburg im Breisgau 79098, Germany

**Keywords:** size-selected clusters, ZnO nanostructures, Schottky junction, chemical bath deposition, Rutherford backscattering spectrometry, transmission electron microscopy, photoluminescence

## Abstract

Hybrid nanostructures combining semiconductor materials and noble metal clusters of atoms (nanoparticles) are of high interest in the energy sector and catalysis, with the idea of tuning the physicochemical properties of the system toward the desired performance. The design of this type of complex system requires the appropriate selection of the material combination to optimize the desired properties. However, less attention has been devoted to the effect of cluster size. In this work, we investigate the size and density effects for mass-selected monometallic Au clusters decorating ZnO-based nanostars. The Au clusters were prepared with narrow control of their size, in terms of atoms per cluster, via cluster deposition in a vacuum and mass selection with a cluster beam source. We study the coupling of ZnO nanostars with deposited Au_*N*_ (*N* = 55, 147, and 309) clusters. We exploit transmission electron microscopy and Rutherford backscattering spectrometry for the structural characterization and for the determination of Au cluster density, obtaining 3.43 × 10^12^, 4.55 × 10^11^, and 7.98 × 10^10^ clusters/cm^2^ for samples decorated with Au clusters containing 55, 147, and 309 atoms, respectively. Moreover, we highlight the formation of a Schottky junction by performing photoluminescence investigations. We find distinctive changes in the behavior of the visible and UV emission as a function of the cluster size and density on the ZnO-based nanostars, identifying an increase of the photoluminescence efficiency with the decrease of the cluster dimension. Our findings indicate the enormous potential that a proper selection of cluster size offers in the fabrication of nanocomposite materials with precise electronic properties.

## Graphical abstract

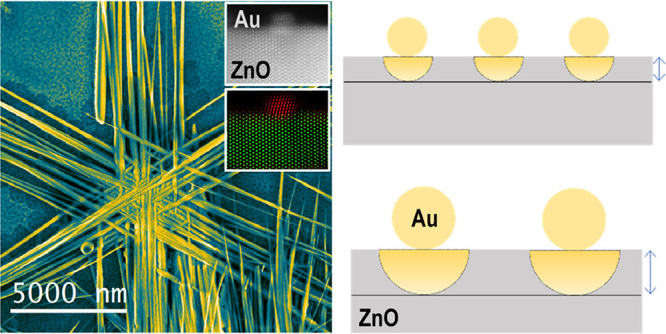

## Introduction

Semiconducting metal oxides are well-known for their significant contribution in various technological application fields, such as solar cells, catalysis, field-effect transistors, and optoelectronic and photoluminescent devices.^1–3^ Group II–VI nanostructured semiconductor materials are of high interest in the fields mentioned above. Among these, zinc oxide (ZnO) stands out thanks to its peculiar physical and chemical properties: it has a direct wide energy band gap (*E*_g_ = 3.2 eV),^4^ it is earth-abundant, nontoxic, and can be fabricated into devices by low-cost and facile preparation processes, yielding a wide array of shapes and morphologies.^4,5^ Indeed, the different ZnO nanostructures have attracted considerable attention, as the material's extrinsic properties can be effectively modulated and regulated by tuning and optimizing its morphology.^6^ In this direction, a novel and promising recent approach is the surface decoration of ZnO with metal nanoclusters (NCs), obtaining a synergistic effect between the inherent properties of the semiconducting oxides and metallic nanomaterials.^7–9^ Typically, ZnO nanostructures exhibit a photoluminescence (PL) spectrum composed of two contributions: the near-band-edge emission in the UV region and a broad luminescent band in the visible range associated with midgap levels induced by oxygen or zinc vacancies.^10,11^ After NC decoration, the visible emission of ZnO is usually enhanced,^9,12,13^ thanks to ZnO band bending at the interface with the metal. This leads to free carrier depletion, which may decelerate the recombination processes. In general, decorating ZnO with metal NCs can lead to an enhancement of the catalytic and electrical properties, as it creates a nano-Schottky junction at the interface between the two materials where a strong electric field is generated.^13^ In this context, different metals have been explored to enhance ZnO properties. For instance, Yao et al.^14^ investigated the effect of Au clusters produced by liquid phase laser ablation on the ZnO properties, discovering that the resulting material presented a higher light absorption and a widened photoresponse. Yoo et al.^15^ discovered that by depositing Ag nanoparticles on ZnO nanorods grown on chemically converted graphene, it is possible to enhance the charge separation, as the complex heterojunctions formed at the interface between the three materials result in the formation of “traps” for photoinduced electrons, causing an increase of the photocatalytic activity of the material for the degradation of methylene blue. Other approaches to coupling ZnO with other metals cause significant modifications of the electrical properties of the resulting material. For instance, Liu et al.^16^ observed that Zn_1–*x*_Mg_*x*_O nanoparticles fabricated via a hydrothermal methodology presented an improved PL response, with the appearance of new emission bands due to the interaction between the surface states of the Mg and of the Zn and because of interstitial vacancies of oxygen. Furthermore, size-selected Au clusters deposited onto ZnO surfaces were found to enhance the activity of the system in CO oxidation^17^ or in various hydrogenation reactions, attributed to active sites at the Au-ZnO perimeter for H_2_ dissociation.^18^

Recently, our group explored the effect of the decoration of ZnO nanorods with monodispersed Au, Pt, and AuPt clusters, obtained via a gas aggregation source (GAS), on the electronic properties of the oxide, observing a strong depletion of the free electrons below the surface of ZnO nanorods.^13^ However, literature outcomes on metal-decorated ZnO nanostructures are conflicting due, on one side, to the difficulties on controlling the nanoclusters’ size and density, and on the other side, to address the cluster size effect onto the heterostructure’s optical and electronic properties. Cluster size and density play a crucial role in defining the physicochemical properties of the heterostructure, as those are locally related to the contact area between metal and semiconductor material. In this work, we shed light into these aspects by exploring the effect of monometallic Au clusters onto ZnO-based nanostars (NSs),^19^ with particular emphasis toward the control of the clusters' size and density, in order to comprehensively clarify the effect of the number of atoms/clusters with respect to the electronic properties of the system. The clusters are produced in a magnetron-based gas aggregation cluster source and mass selected in a lateral time-of-flight (TOF) filter,^20,21^ which ensures monodispersed cluster beams with near atomic precision. These findings could find application in various research fields such as sensing and catalysis.

## Materials and Methods

### ZnO-Based Nanostar Synthesis

ZnO-based NSs were synthesized through chemical bath deposition, starting from an aqueous solution of zinc nitrate and hexamethylenetetramine maintained at 90 °C, to which ammonium fluoride was added to obtain the ZnO NSs containing also a zinc hydroxyfluoride (ZnOHF) phase. A more detailed description of the NS preparation is reported elsewhere.^19^ The resulting nanostructures were washed with Milli-Q water, collected through decantation, and dried in an oven at 100 °C for 24 h. The obtained powders were then dispersed in deionized water.

### Electrode Preparation

Graphene paper (GP) electrodes (1 × 1 cm^2^, 240 μm thick, Sigma-Aldrich, St. Louis, MO, USA) underwent a cleaning process in Milli-Q water followed by drying with N_2_ flux to eliminate impurities for 1 min. NSs were deposited via drop casting using 100 μL of a water dispersion containing 2 mg·ml^–1^ of NSs. The resulting samples were subsequently dried in air.

### Deposition of the Size-Selected Au NC

The Swansea University Nanocluster Source (SUNS), located at the B07 beamline of the Diamond Light Source synchrotron, was used to produce the gold clusters in this study. Size-selected Au clusters were deposited onto ZnO nanostar-coated GP supports and companion transmission electron microscopy (TEM) grids. This DC magnetron-sputtering, inert-gas condensation cluster beam source employed a 2 in. Au target (PI-KEM, 99.99% purity), sputtered with a custom-built magnetron operated at 5 W DC power. Argon flow rates ranged between 200 and 250 sccm, depending on the desired cluster size. The condensation length—the distance from the target face to the exit nozzle of the aggregation chamber—was maintained at 200 mm to facilitate the formation of stable cluster structures. Clusters were formed through condensation of sputtered atoms in a liquid nitrogen-cooled aggregation chamber, with helium flow rates between 50 and 100 sccm (also size-dependent). Prior to deposition, the positively charged cluster ion beam was mass-selected via a lateral TOF mass selector,^20,21^ operating at a mass resolution *M*/Δ*M* of 25, which was calibrated using an Ar^+^ ion beam. The selected Au cluster sizes are 55, 147, and 309 atoms/cluster. Deposition was carried out through a mask containing 3 mm diameter holes in the central region for the GP and TEM supports, corresponding to a deposition area of 0.071 cm^2^ per sample, to achieve uniform cluster coverage of approximately 1% by projected surface area, as measured by beam current. Clusters were soft-landed^22^ on all samples (kinetic energy of 0.5 eV/atom) to minimize damage on landing. The beam and mask holes were aligned to ensure that Gaussian deposition profiles were centered on the sample surfaces.

### Au-Decorated NSs Characterization

NS surface morphology analysis was conducted using a scanning electron microscope (SEM) (Gemini field emission SEM model Carl Zeiss SUPRA 25 from Carl Zeiss Microscopy GmbH in Jena, Germany). SEM images were analyzed by using Digital Micrograph Software to enhance the image brightness and contrast as well as to modify the color palette. Atomic resolution aberration-corrected high-angle annular dark field scanning transmission electron microscopies (AC-HAADF-STEM) were performed in a double aberration-corrected Thermo Fisher Spectra 300 STEM operated at 200 keV. The Spectra 300 is equipped with a Super X energy dispersive X-ray spectroscopy (EDS) detector. For HAADF-STEM and iDPC-STEM, a convergence semiangle of 20 mrad was used.

Rutherford backscattering spectrometry (RBS) was utilized to assess the amount of Au on the electrodes, employing a 2.0 MeV He^+^ beam at normal incidence. All RBS analyses were carried out using a 165° backscattering angle with a 3.5 MV HVEE Singletron accelerator system from High Voltage Engineering Europa. Subsequently, the RBS spectra were analyzed using the XRump software.^23^

PL measurements were conducted by exciting samples with the 325 nm (3.81 eV) line of a He–Cd laser modulated through an acousto-optic modulator at a frequency of 55 Hz. The PL signal was analyzed using a single grating monochromator, detected by a Hamamatsu visible photomultiplier, and measured with a lock-in amplifier having the acousto-optic modulator frequency as a reference.

RBS and PL measurements were carried out for each sample on both bare and Au-decorated ZnO NSs.

## Discussion

The ZnO-based NSs, deposited onto GP substrates and Cu TEM grids, have been decorated with the same ultraprecise Au clusters by placing the grids and substrates in front of the mass-selected Au cluster beam. Three different cluster sizes have been selected: 55 atoms/cluster, 147 atoms/cluster, and 309 atoms/cluster.

Figure 1a reports a representative low-magnification SEM image of NSs, from which the characteristic NS morphology is clearly visible: each nanostructure presents six arms equally spaced within the plane, with each arm composed of bundles of wires. Figure 1b shows a high-magnification SEM image of a nanostar arm decorated with Au with 309 atoms/cluster; from now on, we will refer to this as Au_309_ (similarly will be done for the other samples). The cluster presence is indicated by the white arrows, and it is highlighted by using a blue-yellow color palette. As we can observe, the Au clusters are homogeneously distributed onto the NS branch. In Figure 1c, the high angle annular dark field image obtained in scanning transmission electron microscopy mode (STEM-HAADF) is shown; here, it is possible to clearly observe the Au clusters decorating both the ZnO nanostar branches and the TEM support. The clusters are visible, thanks to the image contrast that, for this type of image, varies as *Z* ^∼2^; therefore, heavier atoms (Au) are brighter, and lighter atoms (Zn, O, and C-from the support) appear darker.^24^ By using STEM-HAADF images, we could perform cluster diameter statistical analysis, as reported in Figure S2 and Table S1.

**Figure 1:**
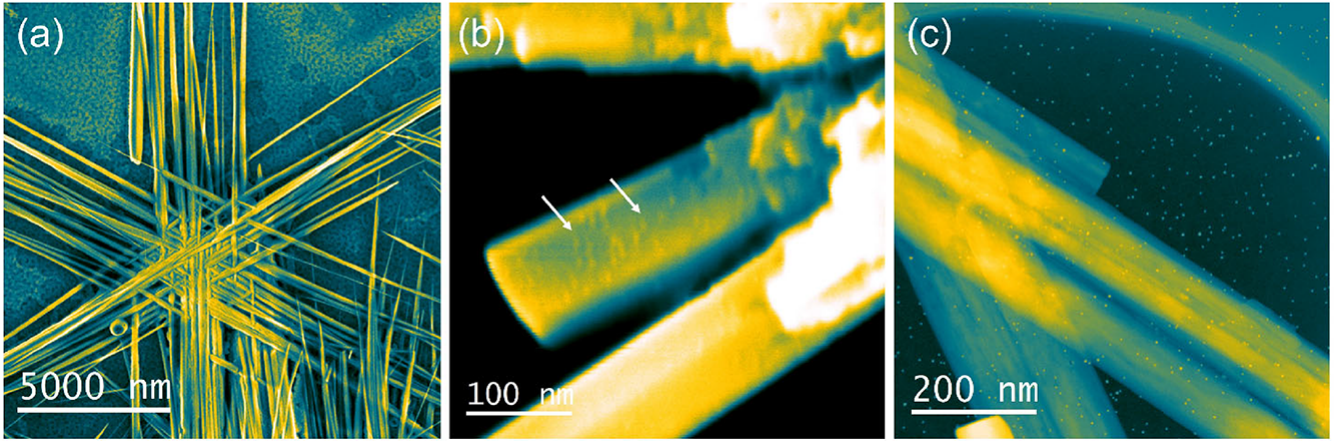
(a) False-colored, low-magnification NS SEM image; (b) false-colored, high-magnification SEM image of an Au_309_-NS branch; and (c) STEM-HAADF image of an Au_309_-NS branch. The images are in a blue-yellow color palette in order to evidence the presence of decorating Au clusters.

A small proportion of the clusters are observed to be larger, corresponding to multiples of Au309 clusters. This observation, seen as a rapidly diminishing set of peaks in the size distribution, when plotting a histogram of measured cluster size from the images, may arise from diffusion and aggregation on the surface after deposition or from multiply charged clusters passing through the mass filter. However, the contribution of this minor proportion of larger clusters is considered negligible in terms of the statistical cluster-size-dependent photoemission relationship found in this study.

By doing an investigation with higher detail of the ZnO NSs branches, it has been possible to retrieve the atomic arrangement of Zn and O. In the first panel of Figure 2, STEM-HAADF (a), Annular Bright Field (STEM-ABF) (b), and Integrated Differential Phase Contrast (STEM-iDPC) (c) micrographs are reported. As we can observe, in Figure 2a, only Zn atoms are visible due to the peculiar contrast of this type of imaging mode.^25^ In Figure 2b,c, both Zn and O atoms are visible: in STEM-ABF, there is an inversion of contrast with the atomic columns shown as dark spots, where the darkest corresponds to the heaviest elements. In STEM-iDPC, the atomic columns are represented as bright spots as in STEM-HAADF, but the image contrast scales more linearly than in STEM-HAADF; therefore, both light and heavy atoms can be imaged together.^26^

**Figure 2:**
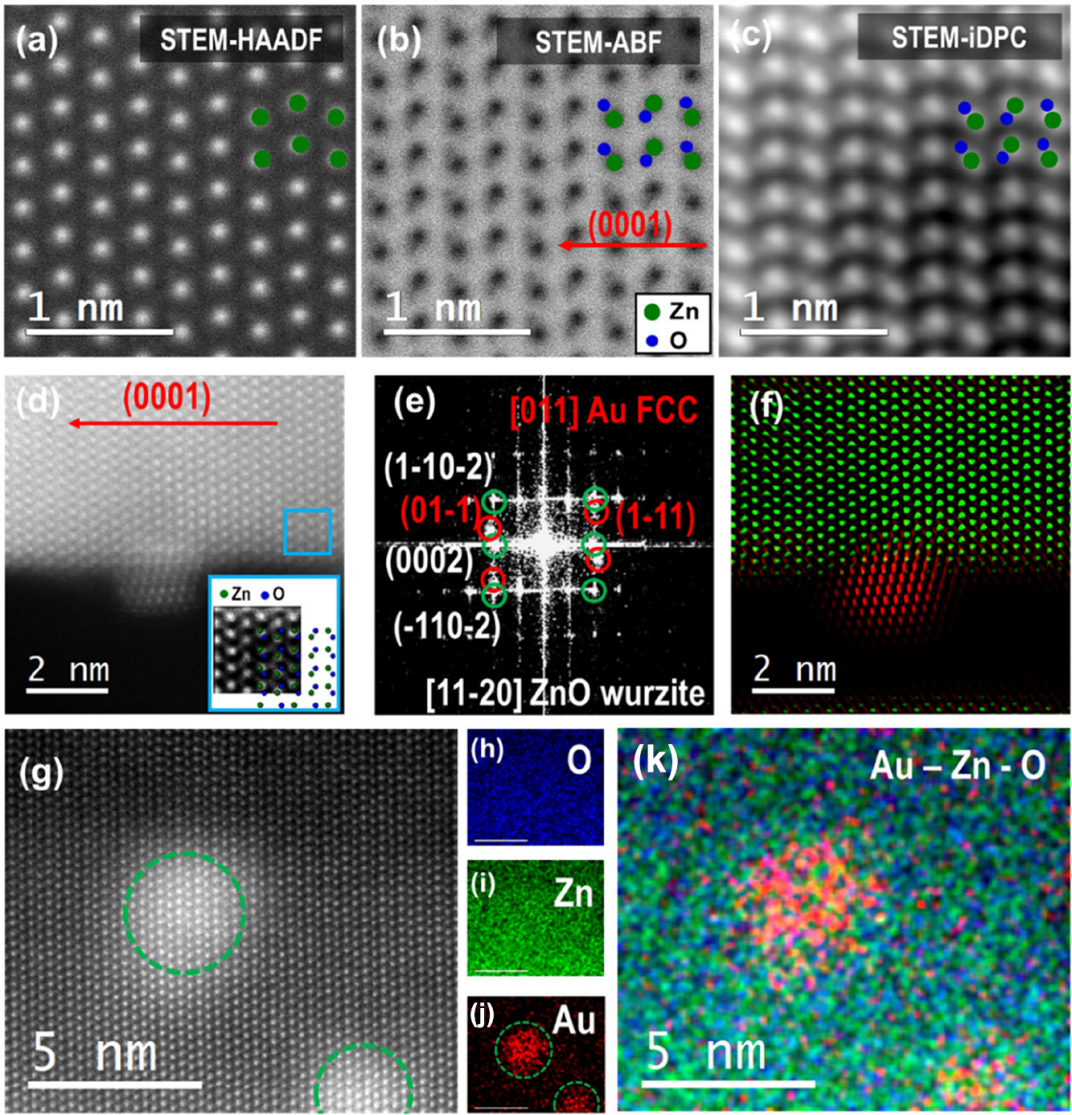
STEM-HAADF (a), STEM-ABF (b), and STEM-iDPC (c) micrographs of ZnO NSs; (d) STEM-HAADF image of the ZnO NS branch decorated with Au55 cluster; (e) power spectrum and (f) frequency filtered map of the same area as in (d). STEM-EDX analysis: (g) STEM-HAADF image of the investigated area, single elemental maps oxygen in (h), Zn in (i), and Au in (j). In (k), the map combining all the elements is reported.

In the central panel of Figure 2, a crystallographic analysis is reported. Here, it has been possible to recognize the power spectra analysis, obtaining that the ZnO possesses a wurzite hexagonal structure and the (0001) growth direction is highlighted, while Au has its cubic structure. By masking and separating the ZnO frequencies from the Au frequencies, it has been possible to construct the frequency-filtered map reported in Figure 2f. Here, it is possible not only to clearly distinguish the ZnO structure from Au, but it is also possible to visualize the different atomic arrangements of the two nanostructured species.

Finally, elemental composition analysis has been performed by STEM-EDX, as reported in the third panel of Figure 2. Here, moving from left to right, it is possible to observe a STEM-HAADF image of the investigated area together with the single element maps and the STEM-EDX map containing all of the elements (Figure 2k). As we can observe, Zn and O are homogeneously distributed within the nanostructure, while Au is localized in the areas of cluster decoration.

The precise chemical composition analysis has been complemented with RBS experiments: in Figure 3a, a schematic of the RBS measurement acquisition condition is reported. The He^2+^ ion beam hits the sample, and the detector is placed at 165° with respect to the beam. Measurements were performed at the center of the sample (inner white circle, “Au”), where the Au clusters are localized, and at the border (outer white circle, “NoAu”), where bare ZnO-based NS are present. Figure 3b shows a representative RBS spectrum, corresponding to Au_147_ samples, acquired in the center and at the edge of the sample (solid and dotted lines, respectively), highlighting the corresponding elements (RBS spectra corresponding to Au_55_ and Au_309_ have been acquired in the same conditions and are reported in Supporting Information, Figure S1). The inset figure is a blowup of the Au peak energy range (1750–1900 eV). As expected, Au appears only in the inner measurements due to the cluster deposition setup. Figure 3c shows the RBS spectra of Au_55_, Au_147_, and Au_309_ samples in the Au peak energy range (1750–1900 eV), demonstrating the different Au content related to the different peak intensity. From the RBS analysis, a highly accurate quantification of the number of Au atoms/cm^2^ is obtained from the peak area, after subtracting the background, as highlighted in Figure 3d. The second column in Table 1 reports the RBS dose for all the electrodes, together with the corresponding error calculated via Poisson statistics: as it is possible to observe, the sample with the smallest clusters’ dimension possesses the highest amount of Au atoms, and also the Au amount decreases with the cluster size. This discrepancy is ascribed to the instability of the cluster beam density during the deposition.

**Figure 3:**
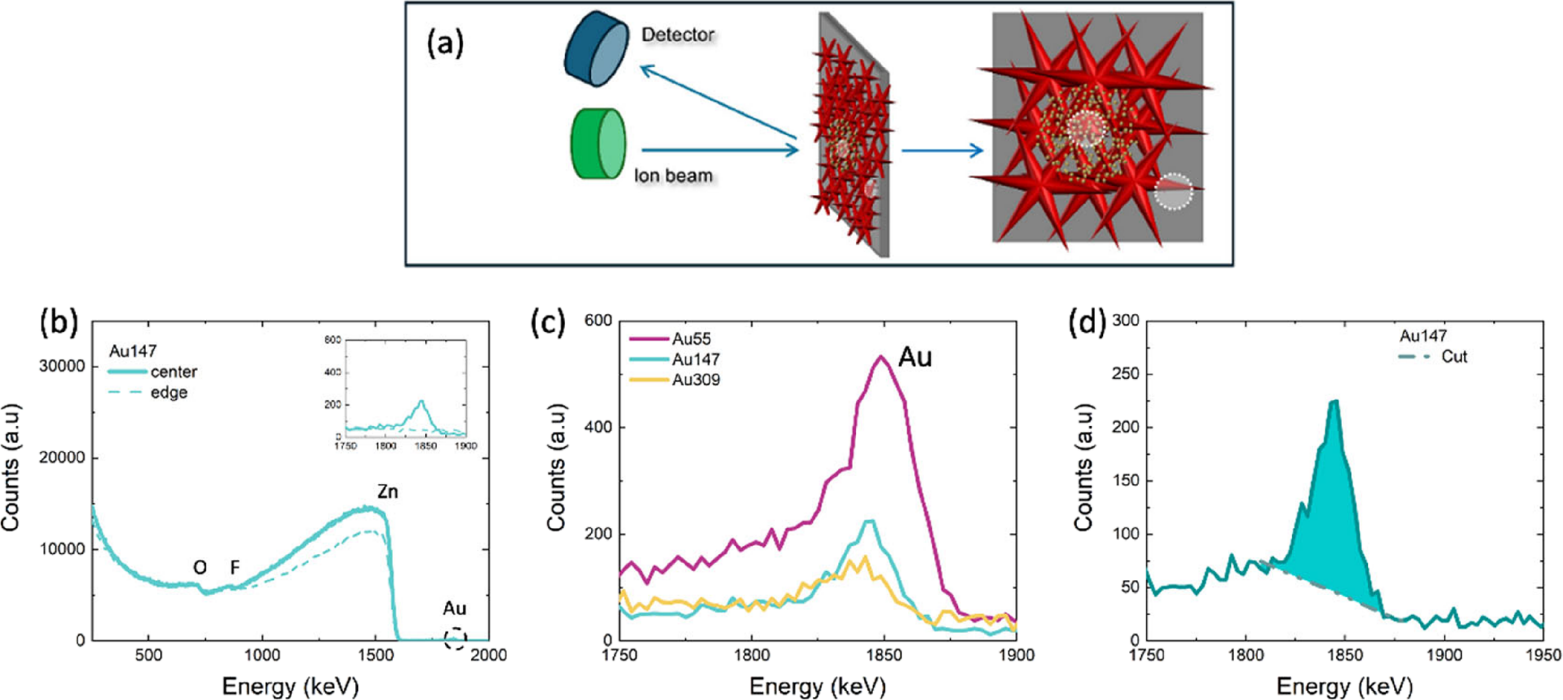
(a) RBS measurements schematic drawing; (b) RBS spectra for Au_147_ sample, made at the center, where the clusters are placed, and at the edge, where the clusters are not deposited (solid and dashed lines, respectively) (c); zoomed Au peak area for Au_55_, Au_147_, and Au_309_ samples; (d) RBS Au peak; the cyan area points out the region considered for the Au atoms calculation.

**Table 1:** RBS Dose and Photoluminescence’s Parameter “η” for All Sample

sample	RBS dose (at cm^–2^)	PL η (decor. ZnO)	PL η (bare ZnO)
Au_55_	3.80 ± 0.07 × 10^14^	3.39	3.02
Au_147_	1.40 ± 0.04 × 10^14^	3.54	3.24
Au_309_	8.30 ± 0.43E13	3.19	2.91

PL spectra were acquired both at the sample center and border to compare bare ZnO with the one decorated with Au in all the samples, considering therefore the different cluster sizes and densities. Figure 4 shows a Jacobian transformation^27^ of the PL spectra of the sample Au_147_ at the sample center (blue) and edge (red) that allows us to show the energy dispersion of the emitted photons. While the UV peak intensity is the same for the two regions considered, the visible band maximum is clearly higher in the presence of Au clusters; this difference is related to the number of photons emitted in the considered energy range.^12^ The ratio between the maximum intensity of the Vis band (*N*_Vis_) and the maximum intensity of the UV band area (*N*_UV_) defines the parameter “η”, which represents the quantity of photons emitted in the visible range per photon emitted in the UV range. Table 1 shows η values for all samples, calculated on both bare and Au-decorated ZnO-based NSs, in order to evidence the net effect of Au cluster decoration, for each selected size. As expected, the η values show small and random variations for bare ZnO, while η systematically increases in the presence of Au. Furthermore, considering each sample individually and comparing the η values for the bare and decorated regions, it is evident that η strongly depends on the number of deposited clusters.

**Figure 4:**
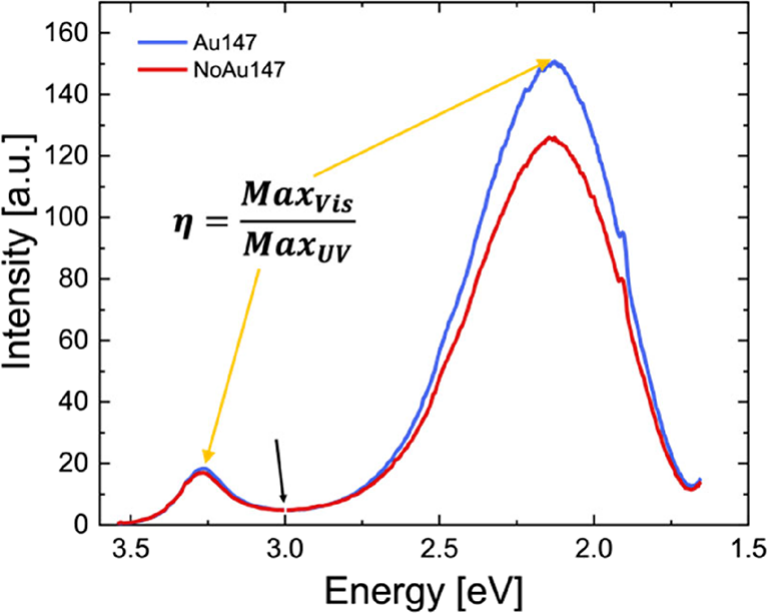
Au_147_ and NoAu_147_ PL spectra. In each spectrum, we have considered 2 regions: UV peak between 3 and 3.6 eV and the Vis peak between 1.6 and 3 eV. The equation reported represents the parameter “η”; Max_Vis_ and Max_UV_ represent the maximum intensity of the Vis band and the maximum intensity of the UV band, as indicated by the arrows.

In order to better visualize the η trend as a function of the cluster size, we calculate Δη = η_Au_– η_no–Au_ obtained by subtracting from the η value of each sample with Au the corresponding η value in the absence of Au clusters. This value was normalized by the density of clusters per cm^2^ (σ), which was calculated by correlating the RBS and TEM data (Au dose and cluster diameter distribution), thus obtaining 3.43 × 10^12^, 4.55 × 10^11^, and 7.98 × 10^10^ clusters/cm^2^ for Au_55_, Au_147_, and Au_309_ samples, respectively (see Supporting Information for details). The corresponding results are reported in Figure 5a for each analyzed sample. As we can observe, $\frac{\Delta \eta }{\sigma }$ varies as a function of the cluster size and progressively increases as the dimension of clusters increases. To describe the observed trend of $\frac{\Delta \eta }{\sigma }$ we proposed a schematic representation of the system in Figure 5b: at the Au (yellow spheres)-ZnO (gray region) interface, a Schottky junction is formed, and ZnO loses electrons that are transferred to Au due to the spillover effect.^13,28^ This leads to a band bending and, consequently, to the formation of a depletion region (light yellow semicircle). This results in the generation of an intense electric field at the Au-ZnO interface (the so-called “halo-effect”), as demonstrated in one of our previous works, through COMSOL software, used to simulate the electric field at the interface between the ZnO and Au nanoparticles. As depicted in Figure 5b, the depletion region depth depends on the projection of Au nanoparticles on ZnO NSs, whereby it increases with the increase of nanoparticle dimensions as demonstrated in ref 13.

**Figure 5:**
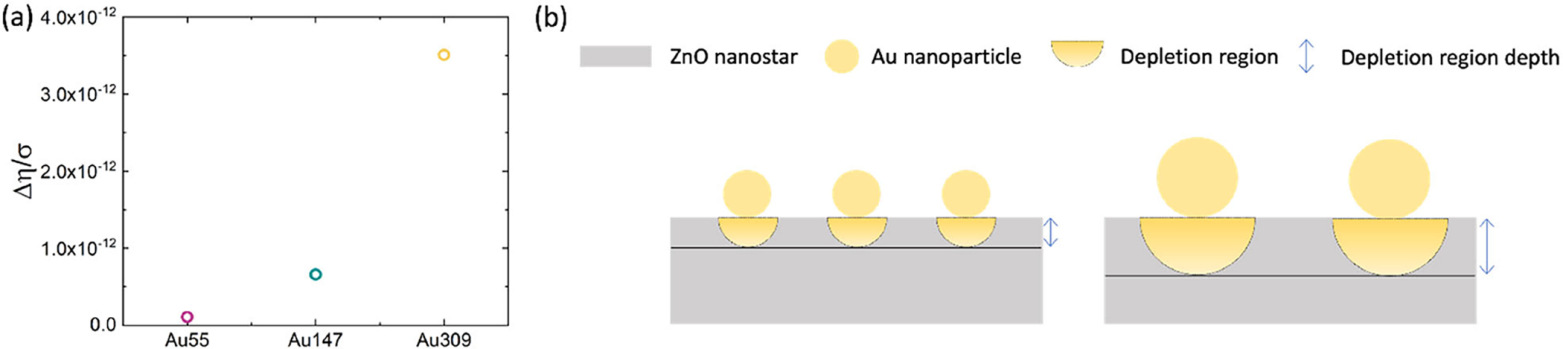
(a) Trend of $\frac{\Delta \eta }{\sigma }$ for each sample; (b) cluster size-effect model. The area below the clusters represents the respective depletion region generated in ZnO by the Au cluster.

This increment in the electric field (or larger depletion region) slows down the electron–hole recombination in the PL process without affecting the exciton transition in the UV range, which is very fast, but promoting radiative pathways in the visible spectrum. A direct consequence is that larger clusters show an enhanced PL effect (higher value of Δη normalized to the cluster density), as shown in Figure 5a.

The effect here described explains the observed PL spectra behavior, shading light into the role of noble metal cluster decoration onto ZnO nanostructured surfaces, with particular emphasis on the cluster size and density.

## Conclusions

In this study, size-selected Au clusters (55, 147, and 309 atoms) were deposited onto the surface of ZnO NSs to comprehensively investigate the effect of the cluster size (in terms of number of atoms per cluster) on the electronic properties of the oxide. We investigated the structural and chemical properties of the hybrid system by performing SEM and aberration-corrected STEM analysis. We observed that the clusters are homogeneously deposited onto the ZnO nanostar surface. We could also recognize the ZnO growth direction, atomic structure, and elemental distribution, the latter investigated with STEM-EDX. These results were complemented with RBS analyses to retrieve the deposited Au density and with PL investigations of the optical properties. The latter showed an enhancement of the visible band intensity as a function of the cluster size and density. This behavior has been ascribed to the Schottky junction formed at the interface between metal and semiconductor material. The effect is the improvement of the charge separation and the photoresponsive behavior of the ZnO nanorods. These data and related discussion open the possibility of using size-selected cluster decoration for fine and local tuning of the electronic properties of this hybrid nanomaterial to contribute to a number of applications with tailored electronic properties.

## Supplementary Material



## Notes

Additional experimental details on the calculation of deposited clusters density combining RBS and TEM results (PDF)
