# Supplementary material for: ZnO Nanostars Decorated with Mass-Selected Au Clusters for Photoluminescence
Source: ACS Appl Nano Mater. 2025 Sep 3;8(36):17463–9. doi: 10.1021/acsanm.5c02913 (PMC12439240; doi:10.1021/acsanm.5c02913)
Supplement: Supplementary file 1 [file an5c02913_si_001.pdf]

Supporting Information

ZnO Nanostars Decorated with Mass-Selected Au Clusters for Photoluminescence

Gisella Di Mari[1,2], Giacometta Mineo[1*], Henry Hoddinott[3,4], Vincenzina Strano[2], Claudio Lentini Campallegio[1,2], Bernat Mundet[5], Sara Martì Sanchez[5], Giorgia Franzò[2], Jordi Arbiol[5,6], Bernd Von Issendorff[7], Georg Held[4], Richard Palmer[3], Elena Bruno[1,2], Salvo Mirabella[1,2] and M. Chiara Spadaro[1,2,5*]

[1] Dipartimento di Fisica e Astronomia "Ettore Majorana", Università di Catania, via S. Sofia 64, Catania 95123, Italy
[2] IMM-CNR, Sede Catania Università, Via S. Sofia 64, 95123 Catania, Italy
[3] Nanomaterials Lab, Mechanical Engineering, Swansea University, Bay Campus, Fabian Way, Swansea, SA1 8EN, UK
[4] Diamond Light Source, Harwell Science and Innovation Campus, Didcot, OX11 0DE, UK
[5] Catalan Institute of Nanoscience and Nanotechnology (ICN2), CSIC and BIST, Campus UAB, Barcelona, Spain
[6] ICREA, Pg. Lluís Companys 23, Barcelona, Catalonia, 08010, Spain
[7] Department of Physics, Albert-Ludwigs-Universität, Freiburg im Breisgau, 79098, Germany

*corresponding authors: giacometta.mineo@dfa.unict.it, mariachiara.spadaro@dfa.unict.it

- **Calculation of deposited clusters density**

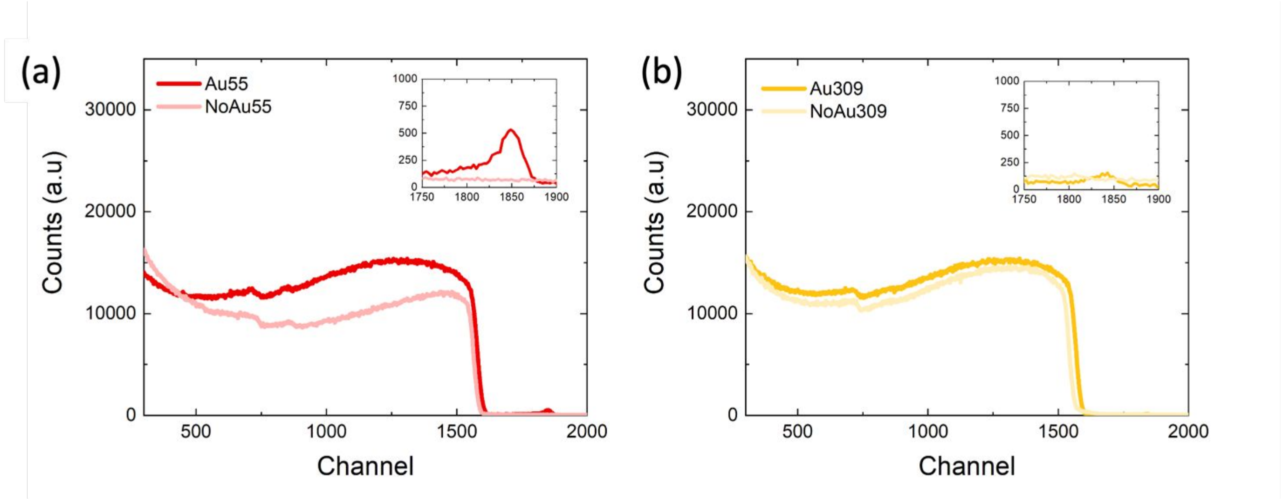

Figure S1: RBS spectra for (a) Au55 and (b) Au309 sample, made at the center, where the cluster are placed and at the edge, where the cluster are not deposited (dark and light line respectively)

Photoluminescence efficiency (η) of Au decorated ZnO samples is correlated with the density of deposited Au clusters, calculated thus combining RBS and TEM results. For all the decorated samples

(Au55, Au147, and Au309) TEM analysis allows to define the clusters diameter distribution (Figure S2 a, c, and e respectively), from which the clusters volume distribution is obtained (Figure S1b, d, and f respectively). Taking into consideration the Au density (19.32 g/cm^3 @ 20 °C, which corresponds to 59.01 at/nm^3), the volume distribution allows to define the mean total number of atoms which are involved in the TEM statistical distribution. The latter, combined with the RBS results concerning the Au dose (at/cm^2), defines the statistic factor, which is an index of the repeatability of the TEM statistical sample per unit area. The number of clusters deposited per unit area is calculated taking into consideration the number of analyzed clusters (253, 369 and 298 for the Au55, Au147, and Au309 samples respectively) and the statistical factor. All the obtained results are reported in Table S1

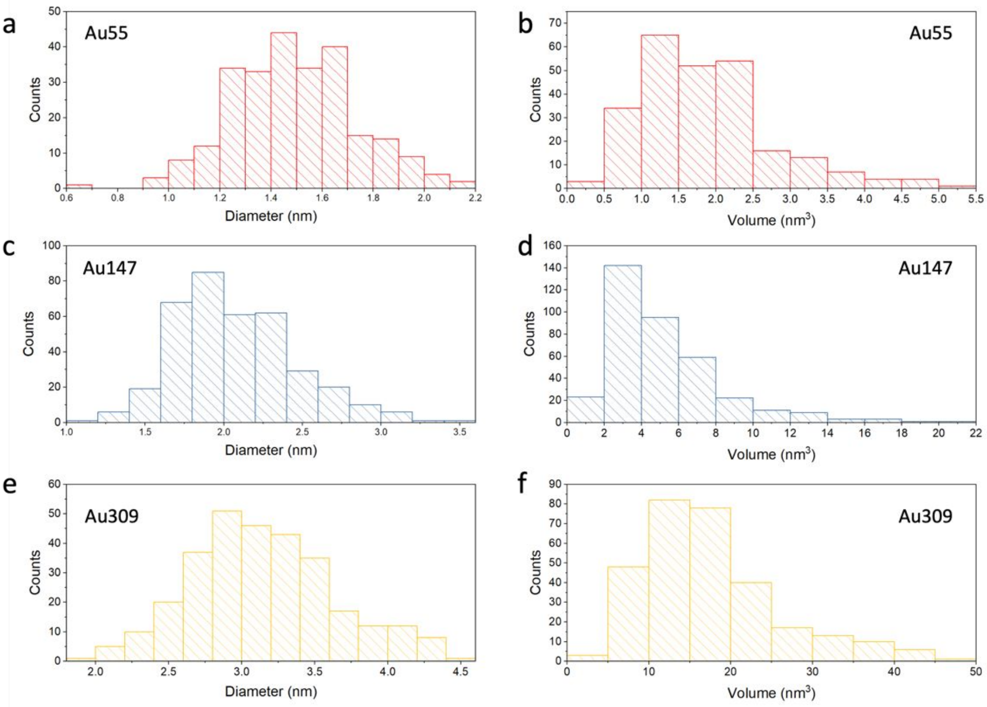

Figure S2: Clusters diameter (a, c, e) and volume (b, d, f) TEM distribution of Au55, Au147 and Au309 samples respectively.

	Average diameter (nm)	Average volume (nm^3)	Dose (at/cm^2)	Statistic Factor	Density of Au clusters (cm^{-2})
**Au55**	1.49	1.86	4.63E14	1.36E10	3.43E12
**Au147**	2.08	5.2	1.5E14	1.23E9	4.55E11

**Au309**	3.15	17.55	1.3E14	2.67E8	7.98E10

Table S1: statistic parameters related to Au55, Au147 and Au309 samples obtained from TEM and RBS analysis, which allow to define the density of Au clusters.
